# Vitamin D receptor polymorphisms in spontaneous preterm birth: a case-control study

**DOI:** 10.3325/cmj.2020.61.338

**Published:** 2020-08

**Authors:** Milena Gašparović Krpina, Anita Barišić, Ana Peterlin, Nataša Tul, Saša Ostojić, Borut Peterlin, Nina Pereza

**Affiliations:** 1 Department of Gynecology and Obstetrics, Clinical Hospital Centre Rijeka, Rijeka, Croatia; 2 Department of Medical Biology and Genetics, Faculty of Medicine, University of Rijeka, Rijeka, Croatia; 3 Clinical Institute for Genomic Medicine, UMC Ljubljana, Ljubljana, Slovenia; 4 Division of Obstetrics and Gynecology, Department of Perinatology, UMC Ljubljana, Ljubljana, Slovenia

## Abstract

**Aim:**

To evaluate the association between the FokI (rs2228570), ApaI (rs7975232), Bsml (rs1544410), TaqI (rs 731236), and Cdx2 (rs11568820) single nucleotide polymorphisms (SNPs) in the vitamin D receptor (*VDR*) gene and spontaneous preterm birth (SPTB), as well as their effect on clinical characteristics of women with SPTB and their newborns.

**Methods:**

This case-control study enrolled women who gave birth at the Department of Obstetrics and Gynecology, University Medical Center Ljubljana between 2010 to 2019. Cases were 118 women with spontaneous initiation of PTB after natural conception and 119 controls with a term singleton delivery after an uncomplicated pregnancy. The molecular analysis of *VDR* SNPs employed polymerase chain reaction and restriction fragment length polymorphism.

**Results:**

Patients and controls did not significantly differ in the distribution of genotype or allele SNP frequencies. However, the FokI polymorphism had a significant effect on newborn birth weight in women with SPTB but not in controls (F = 5.17, *P* = 0.007, one-way ANOVA with *post-hoc* Scheffe test), with newborns of FokI TT carriers having the lowest birth weight (*P* = 0.011). No other *VDR* SNP was associated with any other clinical characteristic of women with SPTB and their newborns.

**Conclusion:**

The TT genotype of the *VDR* FokI polymorphism is associated with newborn birth weight in women of European origin with SPTB.

Preterm birth (PTB), defined as a birth at less than 37 weeks of gestational age (1), represents the leading determinant of neonatal morbidity and mortality, as well as the main cause of long-term neurologic disabilities in children (2). In spite of the progression in obstetrics and clinical management, PTB incidence in the last decades has not been reduced (3).

PTB can be either spontaneous (SPTB) or medically induced. The heterogeneity of SPTB may be attributed to multiple factors, including socioeconomic status, environmental factors, and genetics (4). The etiology of PTB is still not completely understood, although numerous risk factors have been recognized: previous PTB, uterus and cervix anomalies, repeated miscarriages in the second trimester, *in-vitro* fertilization, multiple pregnancy, vaginal bleeding, abnormal placental factors, low socioeconomic status, smoking, etc (5). Considering that personal and family history of PTB are strongest risk factors for a subsequent PTB, research is focused on identifying variability in potential gene candidates (6,7).

The function of vitamin D metabolism in pregnancy, especially PTB, is currently being intensively investigated (8,9). Vitamin D is a fat-soluble vitamin and hormone precursor, with one of its two forms, cholecalciferol (or vitamin D3), being synthesized in the skin by the exposure to UV radiation. The role of vitamin D in calcium and phosphorus metabolism is well known, but in recent years it has been studied in many other biological processes, particularly in immunomodulation at the feto-maternal interface.

Vitamin D deficiency is related to numerous pregnancy complications (10,11). In addition, several studies clearly showed a causal association between vitamin D status in pregnant women and PTB (12-14). Likewise, preterm infants had lower circulating levels of vitamin D (15).

The association between vitamin D levels and PTB may be mediated by single nucleotide polymorphisms (SNPs) in the vitamin D receptor gene (*VDR*) (16,17). Sixty-three polymorphisms of *VDR* gene have been found, and five of them have been intensively investigated: Cdx2 in the promotion region, FokI in the second coding exon, and BsmI, ApaI, and TaqI in the 3′ untranslated region (18). Available evidence indicates a possible causal connection between certain SNPs and PTB, although results are contradictory (16,17,19-21).

Therefore, the aim of our study was to evaluate the association between the FokI (rs2228570), ApaI (rs7975232), Bsml (rs1544410), TaqI (rs 731236), and Cdx2 (rs11568820) SNPs of the *VDR* gene and SPTB, as well as their effect on clinical characteristics of women with SPTB and their newborns.

## PATIENTS AND METHODS

### Patients

This case-control study enrolled women who gave birth at the Division of Perinatology, Department of Obstetrics and Gynaecology, University Medical Center in Ljubljana, Slovenia between 2010 and 2019. All participants gave written informed consent for study participation. The study was approved by the Slovenian National Medical Ethics Committee and the Ethics Committee for Biomedical Research of the Faculty of Medicine, University of Rijeka.

The patient group comprised 118 women with SPTB. Demographic and clinical data of women with SPTB and their newborns, including family history of SPTB, smoking, maternal age, gestational week of birth, and birth weight of newborns, were collected as described in genetic epidemiology studies on PTB (22) by means of a self-developed interviewer-administered questionnaire. The inclusion criteria for the SPTB group were singleton pregnancy following natural conception and spontaneous initiation of PTB before the 37th week of gestation. Gestational age was estimated from the date of last menstrual period and confirmed by ultrasound in the first trimester. If the measurements differed for more than seven days, gestational age was modified according to the ultrasound measurement. The control group consisted of 119 women who had a term birth of a singleton baby after an uncomplicated pregnancy and were of the same age and parity as patients. The exclusion criteria for both patients and controls were diabetes, hypertension, kidney disease, autoimmune conditions, allergic diseases, birth canal infections, *in vitro* fertilization, and complications of pregnancy and congenital anomalies or evidence of infection in newborns. Cases’ and controls’ maternal and newborn characteristics are shown in Table 1.

**Table 1:** Characteristics of patients with spontaneous preterm birth (SPTB) and controls

	No. (%) of			
Maternal characteristics	patients*	controls^†^		P
Mean age at delivery (years)^‡^	30 (18-41)		30 (23-41)	0.385^§^	
Gestational age at delivery			-		
Extremely preterm <28 week	7 (6.2)				
Very preterm 32-28 weeks	33 (29.2)				
Moderate to late preterm 32-37 weeks	73 (64.6)				
Smoking before pregnancy				<0.001^¶^	
yes	37 (32.7)		6 (8.8)		
no	76 (67.3)		62 (91.2)		
Previous PTB			-		
yes	13 (11.5)				
no	100 (88.5)				
Familial PTB			-		
yes	35 (31.8)				
no	75 (68.2)				
**Newborn characteristics**
Mean birth weight (g)^‡^	2334 (605-3915)		3402 (1570-4350)	<0.001^§^	
Congenital anomalies	0		0		
Evidence of infection	0		0		

*available for 113/118 women and newborns with SPTB.

†available for 68/119 control women and newborns.

‡data are presented as mean and range.

§t-test.

¶χ^2^ test.

### DNA extraction and genotyping

Genomic DNA was extracted from peripheral blood leukocytes by standard procedure with Qiagen FlexiGene DNA kit (Qiagen GmbH, Hilden, Germany). All samples were stored at -20 °C. The molecular analysis of *VDR* SNPs employed polymerase chain reaction (PCR) and restriction fragment length polymorphism (RFLP). Genotyping conditions, including primer sequences, PCR protocol, and RFLP fragment sizes are shown in Table 2 (23). PCR reactions were carried out in thermal cyclers (Mastercycle Personal, Eppendorf, Hamburg, Germany and 2720 Thermal Cycler, Applied Biosystems, Carlsbad, CA, USA). Following restriction enzyme digestion, the fragments were separated by electrophoresis on 3% agarose gels stained with GelRed^TM^ (Olerup SSP^®^, Saltsjöbaden, Sweden).

**Table 2:** Genotyping conditions for vitamin D receptor single nucleotide polymorphism (*VDR* SNPs)

*VDR* SNP	Primers	Polymerase chain reaction (PCR) conditions	Fragment sizes (bp)	Restriction enzyme*
**ApaI** **(rs7975232)**	5′-GGATCCTAAATGCACGGAGA-3′ 5′-ACGTCTGCAGTGTGTTGGAC-3′	1. 1 × : 95 °C (5 min) 35 × : 2. 95 °C (60 s) 3. 55 °C (60 s) 4. 72 °C (60 s) 5. 1 × : 72 °C (10 min)	PCR product: 265 T allele: 265 G allele: 146 + 119	ApaI
**BsmI** **(rs1544410)**	5′-CCTCACTGCCCTTAGCTCTG-3′ 5′-TGCCTCCAAAATCAATCAGG-3′		PCR product: 247 A allele: 247 G allele: 144 + 103	BsmI
**Cdx2** **(rs11568820)**	5′-GGATCCCAAAAGGAAAGGAA-3′ 5′-TGTTCCAGATGGTAAAAATAGAATGA-3′		PCR product: 396 A allele: 316 + 80 G allele: 265 + 80 + 51	HpyCH4III
**TaqI** **(rs731236)**	5′-CAGAGCATGGACAGGGAGCAA-3′ 5′-CACTTCGAGCACAAGGGGCGTTAGC-3′		PCR product: 501 T allele: 495 + 6 C allele: 294 + 201 + 6	TaqI
**FokI** **(**rs2228570)	5′-AGCTGGCCCTGGCACTGACTCTGCTCT -3′ 5′-ATGGAAACACCTTGCTTCTTCTCCCTC -3′	1. 1 × : 94 °C (5 min) 35 × : 2. 94 °C (35 s) 3. 61 °C (35 s) 4. 72 °C (1 min) 5. 1 × : 72 °C (7 min)	PCR product: 267 C allele: 267 T allele: 195 + 72	FokI

*New England Biolabs (Ipswich, MA, USA).

### Statistical analysis

Power of the study was calculated with DSS Researcher’s Toolkit (*https://www.dssresearch.com/resources/calculators/statistical-power-calculator-percentage/),* and Hardy-Weinberg equilibrium was calculated with Simple Hardy-Weinberg Calculator – Court Lab (Washington State University College of Veterinary Medicine, Pullman, WA, USA).

The normality of distribution of the numerical variables was tested with the Kolmogorov-Smirnov test. The Pearson chi square test was used to assess the significance of differences in genotype, allele, and haplotype frequencies between the groups. To assess the genetic association with SPTB we calculated odds ratios (OR) and 95% confidence intervals. The *t* test was used to assess the significance of differences in age and newborn birth weight between patients and controls. One-way and two-way analysis of variance (ANOVA) with factorial design and *post-hoc* Scheffe test was used to compare maternal and/or gestational age and fetal birth weight between genotypes of *VDR* SNPs (one-way ANOVA for individual SNPs, two-way ANOVA for the interaction between SNPs). The level of significance was set at 0.05. Statistical analysis was performed with Statistica for Windows, version 12 (StatSoft, Inc., Tulsa, OK, USA) and MedCalc for Windows, version 14.12.0. (MedCalc Software, Mariakerke, Belgium).

## RESULTS

### Genetic association of *VDR* SNPs and SPTB

All genotype frequencies in control women were in Hardy-Weinberg equilibrium (data not shown). The statistical power to detect a 1.5-fold increase in the frequency of the FokI (rs2228570) T allele, ApaI (rs7975232) G allele, Bsml (rs1544410) A allele, Cdx2 (rs11568820), A allele, and TaqI (rs731236) C allele was 90%, 99%, 90%, 90%, and 80%, respectively.

No significant differences were found in the genotype distribution of the *VDR* FokI (rs2228570), ApaI (rs7975232), Bsml (rs1544410), TaqI (rs731236), and Cdx2 (rs11568820) genotype or allele frequencies between women with SPTB and controls (Table 3). No SNP was associated with increased odds for SPTB (Table 3).

**Table 3:** Genotype and allele frequencies of vitamin D receptor single nucleotide polymorphism (*VDR* SNPs) in women with SPTB and controls*

		N (%) of					
*VDR* SNP		women with SPTB	controls	X^2^	P	OR (95% CI)	P
**FokI**	Genotype						
	CC	41 (34.7)	36 (30.2)	1.23	0.540	reference	
	CT	59 (50)	68 (57.1)			1.31 (0.74-2.31)	0.347
	TT	18 (15.2)	15 (12.6)			0.95 (0.42-2.15)	0.900
	Allele						
	C	141 (59.7)	140 (58.8)	0.01	0.912	reference	
	T	95(40.3)	98 (41.2)			1.04 (0.72-1.50)	0.838
**ApaI**	Genotype						
	TG	63 (53.4)	63 (52.9)	0.44	0.803	reference	
	GG	30 (25.4)	34 (28.6)			1.13 (0.62-2.07)	0.684
	TT	25 (21.2)	22 (18.5)			0.88 (0.45-1.72)	0.709
	Allele						
	T	113 (47.9)	157 (65.9)	2.03	0.155	reference	
	G	123 (52.1)	131 (55.0)			0.77 (0.54-1.08)	0.131
**Bsml**	Genotype						
	GG	47 (39.8)	45 (37.8)	0.41	0.813	reference	
	AG	58 (49.1)	63 (52.0)			1.13 (0.66-1.95)	0.788
	AA	13 (11.0)	11 (9.2)			0.88 (0.36-2.17)	0.648
	Allele						
	A	152 (64.4)	153 (64.3)	0.00	0.945	reference	
	G	84 (35.6)	85 (35.7)			1.00 (0.69-1.46)	0.978
**TaqI**	Genotype						
	TT	53 (44.9)	56 (47.0)	0.84	0.655	reference	
	CT	52 (44.0)	54 (45.4)			0.98 (0.57-1.68)	0.949
	CC	13 (11.0)	9 (7.6)			0.65 (0.26-1.66)	0.372
	Allele						
	T	158 (66.9)	166 (69.7)	0.31	0.578	reference	
	C	78 (33.0)	72 (30.2)			0.88 (0.60-1.29)	0.512
**Cdx2**	Genotype						
	GG	80 (67.8)	84 (70.6)	0.61	0.737	reference	
	AG	33 (27.9)	32 (23.9)			0.92 (0.52-1.64)	0.786
	AA	5 (4.2)	3 (2.5)			0.57 (0.13-2.47)	0.454
	Allele						
	G	193 (81.8)	200 (84.0)	0.28	0.596	reference	
	A	43 (18.2)	38 (15.9)			0.85 (0.53-1.38)	0.515

*CI – confidence interval; OR – odds ratio; SNP – single nucleotide polymorphism; SPTB – spontaneous preterm birth.

### Association of clinical characteristics of women and their newborns with *VDR* SNPs

One-way ANOVA showed a significant effect of the FokI and ApaI SNP on newborn birth weight in women with SPTB (FokI F = 5.17, *P* = 0.007; ApaI F = 3.48, *P* = 0.034) but not in the control group (FokI F = 0.57, *P* = 0.569; ApaI F = 0.37, *P* = 0.689). However, two-way ANOVA with factorial design showed no significant interaction between the FokI and ApaI SNPs and found only a significant effect of FokI had on newborn birth weight in women with SPTB (Table 4). *Post-hoc* Scheffe test showed that new-borns of FokI TT carriers had the lowest birth weight (*P* = 0.011) (Table 5). No other *VDR* SNP was associated with any other clinical characteristic of women with SPTB and their newborns.

**Table 4:** Effect of the *VDR* FokI and ApaI gene polymorphisms on newborn birth weight in women with SPTB (two-way ANOVA with factorial design)*

*VDR* SNP	SS	df	F	p
Fok I	3636917	2	4.962	0.009
Apa I	828327	2	1.130	0.327
FokI*ApaI	2490446	4	1.699	0.156

*ANOVA – analysis of variance; SNP – single nucleotide polymorphism; SPTB – spontaneous preterm birth; *VDR* – vitamin D receptor.

**Table 5:** Effect of the *VDR* FokI genotypes on newborn birth weight in women with SPTB (one-way ANOVA with *post-hoc* Scheffe test)*

FokI genotype	Mean birth weight (g)	P
CC	2562.3	0.089^†^
CT	2273.9	0.312^‡^
TT	2009.4	0.011^§^

*ANOVA – analysis of variance; SNP – single nucleotide polymorphism; SPTB – spontaneous preterm birth; *VDR* – vitamin D receptor.

†CC vs CT.

‡CT vs TT.

§TT vs CC.

## DISCUSSION

In this study, TT genotype of the *VDR* FokI polymorphism was associated with newborn birth weight women of European origin with SPTB.

Complex molecular and biological interactions between the mother and fetus are not entirely elucidated. Although newborn birth weight is affected by numerous sociological and environmental factors, the strongest effect is exerted by genetics (24). In addition, a causal relationship between pregnancy outcome and vitamin D has been reported (14,25). Although the role of vitamin D in human growth and bone maturation is well-known, vitamin D plays many other roles during gestation, such as influence on implantation, maintenance of normal pregnancy, support of fetal growth through calcium delivery, secretion of numerous placental hormones, and reduction of proinflammatory cytokine production (25). Given the numerous functions of vitamin D, this study focused on the *VDR* gene as a major etiological factor in SPTB. *VDR* is a mediator of the pleiotropic actions of vitamin D, and its reduced placental expression is implicated in the pathology of complicated pregnancies (26). *VDR* expression in the human placenta indicates that vitamin D could locally influence the fetal-placental development, growth, and cell differentiation as well as signaling at the maternal-fetal interface.

Although the influence of *VDR* SNPs on VDR protein function and signaling is still largely unclear, some polymorphisms affect the circulating levels of vitamin D and its biological activity (27). Research suggests that *VDR* genotype affects the optimal vitamin D concentration and reduces the incidence of adverse perinatal outcome. Although the present study showed an effect of the FokI TT genotype on newborn birth weight, we did not find any difference in the frequencies of FokI genotypes and alleles between patients and controls. However, Manzon et al (20) and Javorski et al (17) showed that the maternal FokI T allele increased the risk for SPTB, with an OR of 3.32 and 7.49, respectively. The only previous study that investigated the association between *VDR* SNPs and newborn birth weight found, contrary to our results, that the FokI A allele was associated with lower birth weight (19). We assume that the differences between these studies might be attributable to patient and control selection, sample size, ethnicity, sun exposure, and maternal life-style.

The FokI SNP is considered an independent marker in the *VDR* gene since it does not show linkage disequilibrium with other polymorphisms within the gene. It is a T-C SNP with two protein variants: a long (T-allele) and a short form of VDR protein (C-allele). Variations in the protein sequence significantly affect the protein function. Using a cell line of human fibroblasts, it has been shown that the FokI “f” isoform (T allele) exhibits a less active form of endogenous *VDR* (28). Since in this study the TT genotype was associated with lower newborn birth weight, we assume that this is the genetic variant responsible for the lower birth weight of prematurely born children. Furthermore, other authors noticed an interesting effect of fetal *VDR* genotype in pregnant women with a lower vitamin D concentration on anthropometric measures at birth. Morley et al (29) observed that infant sequence variants in the *VDR* gene modified the effect of maternal vitamin D deficiency on infant size at birth. Low vitamin D concentrations were associated with lower newborn birth weight only among infants homozygous for the FokI major allele or among heterozygotes.

The function of other *VDR* SNPs – Bsml, ApaI, TaqI, and Cdx2 is less clear. Although we did not demonstrate a significant association with SPTB for any of these SNPs, they represent good research candidates due to the multiple functions of the *VDR* gene, particularly in immunomodulation (cell proliferation, differentiation, invasion, and apoptosis). Baczynska-Strzecha et al (21) showed that individual maternal TaqI, Bsml, and ApaI SNPs did not increase the risk of PTB, but some of the genotype combinations (BsmI-ApaI-TaqI) were significantly more frequent in women with PTB. Rosenfield et al (16) showed the association between both maternal and neonatal *VDR* variants and PTB. The effect of these variants on newborn birth weight was also shown in several studies, which is contrary to our results. Rosenfield et al (16) showed that ApaI AA homozygotes had a 2-fold increased risk for PTB compared with heterozygotes (OR = 1.97). Furthermore, Javorski et al (17) found a significant difference in Cdx2 allele frequencies between the SPTB and control maternal group, and observed an association between this SNP and newborn birth weight. Children of ApaI heterozygotes also had lower birth weight (30). Swamy et al (31) demonstrated the correlation between some other SNPs in the mother's *VDR* gene and lower newborn birth weight in non-Hispanic black women (31). The differences between our and other studies for all five investigated SNPs might be attributed to population differences, participant selection criteria, and sample size.

Future studies should consider environmental factors for low birth-weight, including smoking. The present study showed no association between *VDR* SNP genotypes and smoking before pregnancy. Nevertheless, previous research indicated that cigarette smoke decreased vitamin D production, which could affect *VDR* expression levels (32,33).

A possible limitation of our study is the analysis of only the mother's genotype. However, previous studies have shown that maternal genotype is the most important factor affecting subsequent PTB (34). Additional limitations could be multiple comparisons and possible false positive findings, which is why the robustness of our conclusions should be confirmed by an independent replication study. Our study’s advantages are the strict screening of patients with SPTB and exclusion of all PTBs that did not meet the standard clinical definition of SPTB, as well as a sufficient statistical power of the research.

In conclusion, our study showed that the *VDR* FokI SNP was associated with SPTB in women of European origin and that it could affect the lower birth weight of their newborns. Nevertheless, other genetic associations and expression studies in different populations are needed to investigate the role of *VDR* SNPs in SPTB, especially studies assessing maternal vitamin D status.

## References

[1] World Health OrganizationPreterm birth. c2019 - [cited 2019 Mar 19]. Available from: https://www.who.int/news-room/factsheets/detail/preterm-birth. Accessed: August 26, 2020.

[2] Shapiro-Mendoza CK, Lackritz EM (2012). Epidemiology of late and moderate preterm birth.. Semin Fetal Neonatal Med.

[3] Beck S, Wojdyla D, Say L, Betran AP, Merialdi M, Requejo JH (2010). The worldwide incidence of preterm birth: a systematic review of maternal mortality and morbidity.. Bull World Health Organ.

[4] Gimenez LG, Krupitzki HB, Momany AM, Gili JA, Poletta FA, Campaña H (2016). Maternal and neonatal epidemiological features in clinical subtypes of preterm birth.. J Matern Fetal Neonatal Med.

[5] Menon R (2008). Spontaneous preterm birth, a clinical dilemma: etiologic, pathophysiologic and genetic heterogeneities and racial disparity.. Acta Obstet Gynecol Scand.

[6] Anum EA, Springel EH, Shriver MD, Strauss JF (2009). Genetic contributions to disparities in preterm birth.. Pediatr Res.

[7] Crider KS, Whitehead N, Buus RM (2005). Genetic variation associated with preterm birth: a HuGE review.. Genet Med.

[8] Qin LL, Lu FG, Yang SH, Xu HL, Luo BA (2016). Does maternal vitamin D deficiency increase the risk of preterm birth: a meta-analysis of observational studies.. Nutrients.

[9] Møller UK, Streym S, Mosekilde L, Heickendorff L, Flyvbjerg A, Frystyk J (2013). Changes in calcitropic hormones, bone markers and insulin-like growth factor I (IGF-I) during pregnancy and postpartum: a controlled cohort study.. Osteoporos Int.

[10] Kiely  M (2017). Hemmingway A2, O’Callaghan KM2. Vitamin D in pregnancy: current perspectives and future directions.. Ther Adv Musculoskelet Dis.

[11] Palacios C, De-Regil LM, Lombardo LK, Peña-Rosas JP (2016). Vitamin D supplementation during pregnancy: Updated meta-analysis on maternal outcomes.. J Steroid Biochem Mol Biol.

[12] Amegah AK, Klevor MK, Wagner CL (2017). Maternal vitamin D insufficiency and risk of adverse pregnancy and birth outcomes: A systematic review and meta-analysis of longitudinal studies.. PLoS One.

[13] Bodnar LM, Klebanoff MA, Gernand AD, Platt RW, Parks WT, Catov JM (2014). Maternal vitamin D status and spontaneous preterm birth by placental histology in the US Collaborative Perinatal Project.. Am J Epidemiol.

[14] Zhou SS, Tao YH, Huang K, Zhu BB, Tao FB (2017). Vitamin D and risk of preterm birth: Up-to-date metaanalysis of randomized controlled trials and observational studies.. J Obstet Gynaecol Res.

[15] Burris HH, Van Marter LJ, McElrath TF, Tabatabai P, Litonjua AA, Weiss ST (2014). Vitamin D status among preterm and fullterm infants at birth.. Pediatr Res.

[16] Rosenfeld T, Salem H, Altarescu G, Grisaru-Granovsky S, Tevet A, Birk R (2017). Maternal-fetal vitamin D receptor polymorphisms significantly associated with preterm birth.. Arch Gynecol Obstet.

[17] Javorski N, Lima CAD, Silva LVC, Crovella S, de Azêvedo Silva J (2018). Vitamin D receptor (VDR) polymorphisms are associated to spontaneous preterm birth and maternal aspects.. Gene.

[18] Uitterlinden AG, Fang Y, Van Meurs JB, Pols HA, Van Leeuwen JP (2004). Genetics and biology of vitamin D receptor polymorphisms.. Gene.

[19] Barchitta M, Maugeri A, La Rosa MC, Magnano San Lio R, Favara G, Panella M (2018). Single nucleotide polymorphisms in vitamin D receptor gene affect birth weight and the risk of preterm birth: results from the “Mamma & Bambino” cohort and a meta-analysis.. Nutrients.

[20] Manzon L, Altarescu G, Tevet A, Schimmel MS, Elstein D, Samueloff A (2014). Vitamin D receptor polymorphism FokI is associated with spontaneous idiopathic preterm birth in an Israeli population.. Eur J Obstet Gynecol Reprod Biol.

[21] Baczyńska-Strzecha M, Kalinka J (2016). Influence of Apa1 (rs7975232), Taq1 (rs731236) and Bsm1 (rs154410) polymorphisms of vitamin D receptor on preterm birth risk in the Polish population.. Ginekol Pol.

[22] Pennell CE, Jacobsson B, Williams SM, Buus RM, Muglia LJ, Dolan SM (2007). Genetic epidemiologic studies of preterm birth: guidelines for research.. Am J Obstet Gynecol.

[23] Hajj A, Chedid R, Chouery E, Megarbané A, Gannagé-Yared MH (2016). Relationship between vitamin D receptor gene polymorphisms, cardiovascular risk factors and adiponectin in a healthy young population.. Pharmacogenomics.

[24] Mallia T, Grech A, Hili A, Pace NP (2017). Genetic determinants of low birth weight.. Minerva Ginecol.

[25] Liu NQ, Hewison M (2012). Vitamin D, the placenta and pregnancy.. Arch Biochem Biophys.

[26] Murthi P, Yong HE, Ngyuen TP, Ellery S, Singh H, Rahman R (2016). Role of the placental vitamin D receptor in modulating feto-placental growth in fetal growth restriction and preeclampsia-affected pregnancies.. Front Physiol.

[27] McGrath JJ, Saha S, Burne TH, Eyles DW (2010). A systematic review of the association between common single nucleotide polymorphisms and 25-hydroxyvitamin D concentrations.. J Steroid Biochem Mol Biol.

[28] Whitfield GK, Remus LS, Jurutka PW, Zitzer H, Oza AK, Dang HT (2001). Functionally relevant polymorphisms in the human nuclear vitamin D receptor gene.. Mol Cell Endocrinol.

[29] Morley R, Carlin JB, Pasco JA, Wark JD, Ponsonby AL (2009). Maternal 25-hydroxyvitamin D concentration and offspring birth size: effect modification by infant VDR genotype.. Eur J Clin Nutr.

[30] Pereira-Santos M, Carvalho GQ, Louro ID, Dos Santos DB, Oliveira AM (2019). Polymorphism in the vitamin D receptor gene is associated with maternal vitamin D concentration and neonatal outcomes: A Brazilian cohort study.. Am J Hum Biol.

[31] Swamy GK, Garrett ME, Miranda ML, Ashley-Koch AE (2011). Maternal vitamin D receptor genetic variation contributes to infant birthweight among black mothers.. Am J Med Genet A.

[32] Banihosseini SZ, Baheiraei A, Shirzad N, Heshmat R, Mohsenifar A (2013). The effect of cigarette smoke exposure on vitamin D level and biochemical parameters of mothers and neonates.. J Diabetes Metab Disord.

[33] Mulligan JK, Nagel W, O’Connell BP, Wentzel J, Atkinson C, Schlosser RJ (2014). Cigarette smoke exposure is associated with vitamin D3 deficiencies in patients with chronic rhinosinusitis.. J Allergy Clin Immunol.

[34] Khoury MJ, Cohen BH, Diamond EL, Chase GA, McKusick VA (1987). Inbreeding and prereproductive mortality in the Old Order Amish. III. Direct and indirect effects of inbreeding.. Am J Epidemiol.

